# Thyroid autoimmunity, thyroid function, and endometriosis: reproductive immune-endocrine crosstalk and causal uncertainty

**DOI:** 10.3389/fmed.2026.1916744

**Published:** 2026-08-07

**Authors:** Shida Chen

**Affiliations:** Department of Endocrinology, The Second People's Hospital of Bengbu City, Bengbu, Anhui, China

**Keywords:** assisted reproduction, causal inference, endometriosis, immune-endocrine crosstalk, infertility, reproductive endocrinology, thyroid autoimmunity, thyroid function

## Abstract

**Background:**

Endometriosis is an estrogen-dependent inflammatory disorder associated with pelvic pain, infertility, repeated treatment, and impaired quality of life. Thyroid dysfunction and thyroid autoimmunity are also common in reproductive-aged women and may affect menstrual cyclicity, ovarian function, implantation, pregnancy maintenance, and assisted reproduction. Observational studies suggest an association between thyroid-related phenotypes and endometriosis, but its directionality, reproductive relevance, and biological basis remain uncertain.

**Main body:**

This narrative review synthesizes epidemiological, mechanistic, reproductive, environmental, genetic, and causal-inference evidence on the thyroid–endometriosis interface. It distinguishes biochemical thyroid function traits, thyroid dysfunction, thyroid autoimmunity, and diagnosis-based thyroid disease as separate but related constructs. Current evidence suggests that thyroid autoimmunity may be more relevant to endometriosis than isolated thyroid hormone abnormalities. Cohort, register-based, case-control, and infertility-population studies have reported associations with subsequent thyroid morbidity, thyroid autoantibody positivity, and assisted reproduction outcomes, although findings remain heterogeneous across populations, study designs, and phenotype definitions. Mechanistic evidence is best interpreted at three levels: established endometriosis biology, indirect thyroid-related reproductive plausibility, and shared upstream susceptibility involving endocrine-disrupting chemicals and genetic or epigenetic vulnerability. Direct thyroid-to-lesion evidence remains limited. Most available evidence is observational. A recent bidirectional Mendelian randomization study reported several phenotype-specific associations, but the estimates require independent replication and do not establish a uniform thyroid-to-endometriosis causal relationship.

**Conclusion:**

The thyroid–endometriosis association is better viewed as a phenotype-specific reproductive immune–endocrine interface than as a simple thyroid hormone-driven causal pathway. Current evidence does not justify universal thyroid screening solely because endometriosis is present. Thyroid evaluation should instead follow established endocrine, reproductive, pregnancy-related, or autoimmune indications, including infertility, recurrent pregnancy loss, menstrual disturbance, planned assisted reproduction, symptoms of thyroid dysfunction, previous abnormal thyroid tests, or coexisting autoimmune disease. Future studies should combine standardized phenotyping, prospective follow-up, mechanistic biomarkers, and causal-inference methods to determine whether thyroid-related pathways are clinically useful markers, modifiable contributors, or parallel manifestations of systemic dysregulation in endometriosis.

## Background

Endometriosis is a chronic gynecological disorder defined by the presence of endometrial-like glands and stroma outside the uterine cavity. It predominantly affects women of reproductive age and is associated with dysmenorrhea, chronic pelvic pain, dyspareunia, infertility, repeated treatment, and impaired quality of life (1, 2). Although retrograde menstruation remains a foundational theory, lesion establishment requires a permissive biological environment involving estrogen-dependent proliferation, progesterone resistance, angiogenesis, neurogenesis, fibrosis, and inflammatory signaling (3, 4). Impaired immune clearance and altered immune regulation may further contribute to the survival and persistence of ectopic endometrial tissue (5, 6).

Thyroid dysfunction and thyroid autoimmunity are also clinically relevant in reproductive medicine. Overt and subclinical hypothyroidism, hyperthyroidism, Hashimoto's thyroiditis, Graves' disease, and isolated thyroid autoantibody positivity may affect menstrual cyclicity, ovulation, ovarian reserve, implantation, pregnancy maintenance, and assisted reproductive technology (ART) outcomes, although the strength of evidence varies by thyroid phenotype and reproductive setting (7–9). Clinically relevant thyroid markers include thyroid-stimulating hormone (TSH), free thyroxine (FT4), free triiodothyronine (FT3), thyroid peroxidase antibodies (TPOAb), thyroglobulin antibodies (TgAb), and TSH receptor antibodies (TRAb) (7, 8). Thyroid hormone abnormalities and thyroid autoimmunity are related but distinct constructs. Autoimmune thyroid disease may occur in euthyroid women, whereas altered thyroid function may occur without detectable thyroid autoantibodies (7, 10).

The intersection between thyroid biology and endometriosis is particularly relevant to reproductive biology and endocrinology because it involves uterine biology, reproductive immunology, endocrine regulation, infertility, and assisted reproduction (1, 4, 10). Cohort and register-based studies have reported increased subsequent risks of thyroid-related morbidity after endometriosis diagnosis (11, 12). Retrospective and cross-sectional studies have suggested higher frequencies of thyroid autoantibody positivity in women with endometriosis (13, 14). However, findings remain inconsistent across populations, study designs, thyroid phenotypes, and endometriosis phenotypes (10, 15). TRAb studies have yielded discordant findings, and the reproductive impact of coexisting endometriosis and thyroid autoimmunity in assisted reproduction remains uncertain (15–17). This inconsistency raises a central question: does thyroid dysfunction contribute to endometriosis pathogenesis, does endometriosis alter thyroid regulation, or do both conditions reflect shared reproductive immune-endocrine susceptibility?

## Evidence identification and synthesis

This article was designed as a critical narrative review rather than a systematic review. Targeted, non-systematic searches of PubMed/MEDLINE were conducted from database inception through July 23, 2026 and were supplemented by screening the reference lists of key reviews and primary studies. Search terms combined endometriosis with thyroid function, thyroid autoimmunity, hypothyroidism, hyperthyroidism, thyroid autoantibodies, infertility, assisted reproduction, inflammation, immune mechanisms, endocrine-disrupting chemicals, genetic susceptibility, and Mendelian randomization (MR). Priority was given to guidelines, systematic reviews, meta-analyses, cohort and register-based studies, infertility-population studies, mechanistic studies, and genetic or causal-inference studies directly relevant to the thyroid–endometriosis interface. Particular emphasis was placed on studies evaluating thyroid-related morbidity after endometriosis and thyroid autoimmunity in infertility (11, 12, 18). Genetic and causal-inference evidence was considered when it addressed shared biological susceptibility or causal interpretation (19–21). Because the purpose was interpretive synthesis rather than effect-size estimation, formal meta-analysis and risk-of-bias grading were not performed.

**Figure 1:**
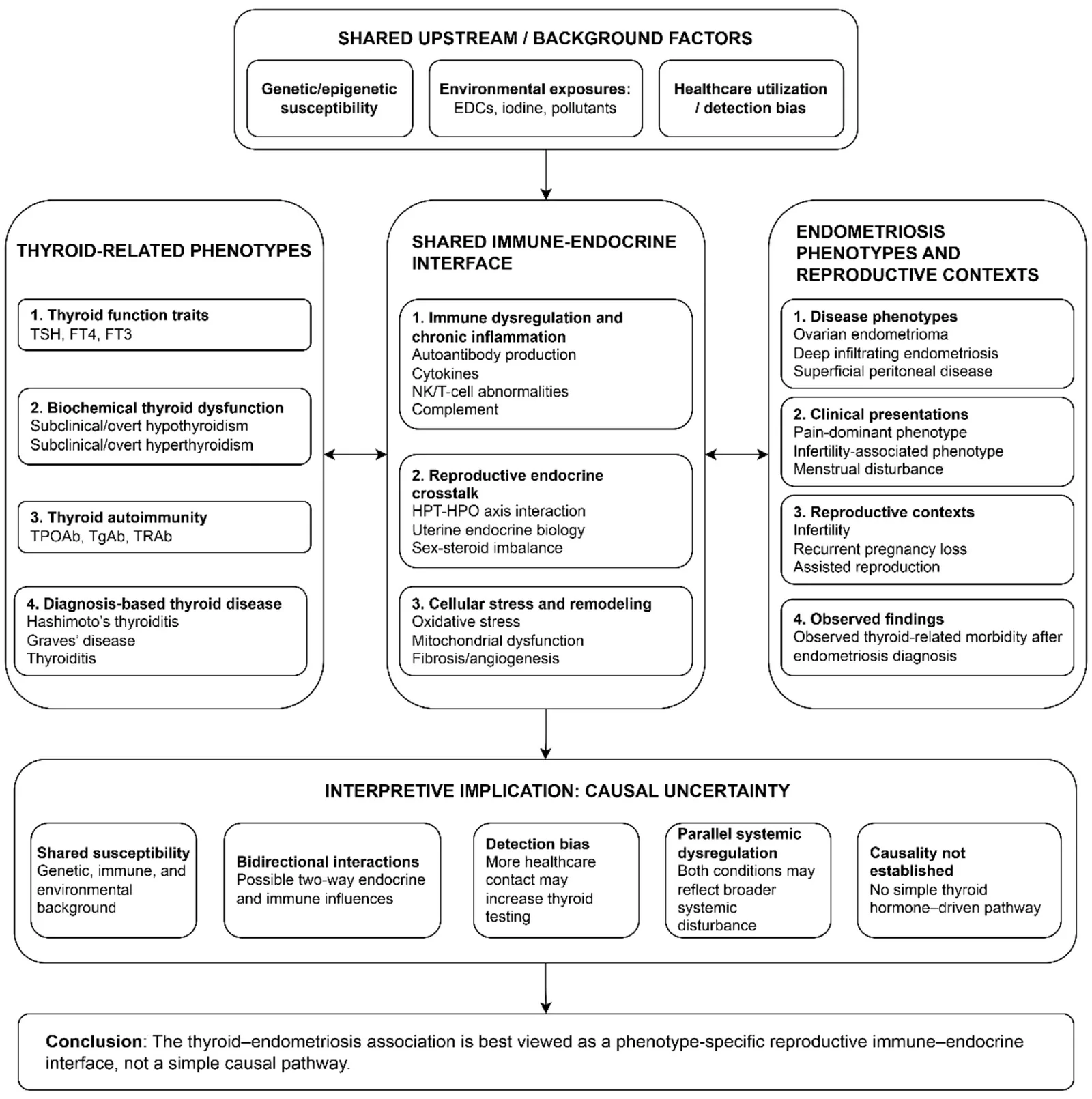
Phenotype-specific reproductive immune–endocrine framework linking thyroid-related phenotypes and endometriosis. This framework summarizes how thyroid-related phenotypes may intersect with endometriosis through shared upstream factors, immune dysregulation, chronic inflammation, reproductive endocrine crosstalk, cellular stress, and tissue remodeling. Thyroid function traits, biochemical thyroid dysfunction, thyroid autoimmunity, and diagnosis-based thyroid disease are presented as related but distinct constructs. The framework supports biological plausibility, shared susceptibility, bidirectional interaction, detection bias, and causal uncertainty, but it does not establish a simple thyroid hormone–driven causal pathway. EDCs, endocrine-disrupting chemicals; FT3, free triiodothyronine; FT4, free thyroxine; HPO, hypothalamic-pituitary-ovarian; HPT, hypothalamic-pituitary-thyroid; TgAb, thyroglobulin antibodies; TPOAb, thyroid peroxidase antibodies; TRAb, TSH receptor antibodies; TSH, thyroid-stimulating hormone.

The aim of this review was to provide a critical synthesis tailored to reproductive biology and endocrinology rather than a general review of thyroid disease or endometriosis alone. Its added value lies in explicitly separating biochemical thyroid function traits, biochemical thyroid dysfunction, thyroid autoimmunity, and diagnosis-based thyroid disease as distinct constructs. The review also integrates recent cohort, register-based, infertility-population, TRAb-focused, genetic-overlap, and causal-inference evidence into a reproductive immune–endocrine framework. This structure shifts the question from whether thyroid disease causes endometriosis to which thyroid-related phenotype may be relevant, in which reproductive context, through which immune, endocrine, environmental, or genetic pathway, and with what degree of causal support. A phenotype-specific conceptual framework for the thyroid–endometriosis interface is shown in Figure 1.

## Thyroid function and thyroid autoimmunity in reproductive biology

Thyroid function is regulated through the hypothalamic-pituitary-thyroid (HPT) axis. TSH promotes thyroid hormone synthesis, while circulating thyroid hormones provide negative feedback to the hypothalamus and pituitary (22). Peripheral conversion of thyroxine (T4) to biologically active triiodothyronine (T3) further complicates the interpretation of serum thyroid markers, particularly in reproductive settings where thyroid function and reproductive outcomes intersect (7, 22).

Clinically, elevated TSH with reduced FT4 indicates overt hypothyroidism, whereas elevated TSH with normal FT4 is generally classified as subclinical hypothyroidism (9, 22). Suppressed TSH with elevated FT4 and/or FT3 indicates overt hyperthyroidism, whereas suppressed TSH with normal thyroid hormone concentrations suggests subclinical hyperthyroidism (23). These categories are useful for clinical classification, but they do not fully capture tissue-level thyroid hormone action, peripheral thyroid hormone metabolism, or context-specific factors that may affect thyroid test interpretation (7, 22).

Thyroid autoimmunity represents a separate dimension of thyroid disease. TPOAb and TgAb are commonly associated with Hashimoto's thyroiditis, whereas TRAb are central to Graves' disease (7, 23). In reproductive medicine, thyroid autoimmunity has been associated with infertility and adverse pregnancy outcomes, even in euthyroid women (7, 18). However, it remains uncertain whether thyroid antibodies act as pathogenic mediators or as markers of broader systemic immune dysregulation (7, 10).

Interpretation of thyroid markers in women with endometriosis therefore requires caution. TSH and thyroid hormone levels may be influenced by age, body mass index (BMI), iodine intake, pregnancy, medication use, systemic illness, assay interference, and inflammatory status (8, 9, 22). Endometriosis-related factors, including chronic inflammation, infertility evaluation, treatment history, and increased healthcare contact, may also influence thyroid testing patterns and diagnostic ascertainment (10–12). For this reason, thyroid hormone abnormalities and thyroid autoimmunity should be interpreted as related but distinct constructs rather than as interchangeable indicators of a single thyroid exposure (7, 10).

## Endometriosis as a reproductive immune-endocrine disease

Endometriosis is increasingly viewed as a chronic systemic inflammatory disorder with reproductive immune-endocrine features, rather than a purely localized pelvic disease (1, 2). Estrogen dependence is a defining feature. Ectopic lesions may show enhanced local estrogen biosynthesis, altered estrogen receptor signaling, and reduced responsiveness to progesterone, thereby supporting lesion survival, inflammation, and resistance to normal cyclic regulation (3, 4).

Progesterone resistance may impair progesterone-responsive endometrial programs, sustain inflammatory signaling, and weaken anti-proliferative control (3, 4). Differences in progesterone receptor expression, estrogen receptor expression, and adhesion-related molecules across endometriosis phenotypes suggest that ovarian endometrioma and deep infiltrating endometriosis are not biologically interchangeable (24, 25).

Immune dysfunction is also central to endometriosis. Altered systemic and uterine immune populations, impaired peritoneal natural killer (NK) cell chemotaxis, inflammatory immune-cell activation, and immune checkpoint involvement have been reported (5, 6, 26). Changes in cytokine profiles and complement-related inflammatory networks may further contribute to lesion persistence and phenotype heterogeneity (27, 28). These abnormalities may allow ectopic endometrial cells to evade immune clearance, persist at ectopic sites, promote angiogenesis, and maintain a chronic inflammatory microenvironment (1, 5, 26).

This immune-endocrine profile provides biological plausibility for overlap between endometriosis and autoimmune thyroid disease, but it does not by itself establish thyroid autoimmunity as a cause of endometriosis (10, 13). This distinction is important because immune overlap, clinical co-occurrence, and shared inflammatory biology do not necessarily imply a direct thyroid-driven causal pathway (10, 13).

## Epidemiological evidence linking thyroid disorders and endometriosis

The epidemiological evidence linking thyroid disorders and endometriosis is suggestive but heterogeneous (10). The most recurrent signal involves thyroid autoimmunity rather than isolated thyroid hormone abnormalities (10, 13, 14). Retrospective and cross-sectional studies have reported higher frequencies of thyroid autoantibody positivity, particularly TPOAb and TgAb, in women with endometriosis (13, 14). One retrospective study also suggested that combining thyroid autoantibodies with established gynecological markers such as cancer antigen 125 (CA125) may improve diagnostic discrimination in selected populations (14). However, these findings remain population-specific and should not be interpreted as evidence that thyroid autoantibodies are clinically established diagnostic markers for endometriosis.

TRAb have also been investigated as possible thyroid-related biomarkers in endometriosis. Early work suggested that TRAb-related autoantibodies may be elevated in women with endometriosis, raising the possibility of an additional autoimmune link (16). However, a subsequent study using current clinical methods did not confirm elevated immunoglobulin G (IgG) TRAb in endometriosis (15). These discrepancies highlight assay-related uncertainty, population differences, and the need for independent validation (15, 16). TRAb should therefore be discussed as a research signal rather than a clinically actionable endometriosis biomarker.

Cohort and register-based studies provide stronger temporal evidence than cross-sectional studies. Long-term observational data suggest that women with endometriosis may have increased subsequent risks of thyroid-related morbidity, including functional thyroid disorders, thyroiditis, and other thyroid diagnoses (11). Register-based studies of surgically verified endometriosis have also supported increased somatic morbidity, including thyroid-related morbidity, after endometriosis diagnosis (12). These data suggest that endometriosis may be associated with later thyroid disease. Nevertheless, residual confounding and surveillance bias remain important concerns (10, 11). Women with endometriosis may have more frequent healthcare contact, infertility evaluation, and long-term follow-up, which could increase the likelihood of thyroid testing and diagnostic ascertainment (10, 11).

Infertility and assisted reproduction populations are especially relevant to reproductive biology and endocrinology. Thyroid autoimmunity is more common in infertile women overall, and endometriosis is a major cause of infertility (2, 18). In women undergoing *in vitro* fertilization (IVF), the coexistence of endometriosis and thyroid autoimmunity has been investigated, but its independent effect on reproductive outcomes remains uncertain (17, 29). Current evidence does not support a simple causal effect of TSH or FT4 on overall infertility (19). However, phenotype-specific findings suggest that broad infertility outcomes may obscure disease-specific relationships involving endometriosis, thyroid autoimmunity, or selected reproductive subgroups (17, 19, 29). A July 2026 bidirectional two-sample MR study reported an inverse association between genetically predicted FT4 and overall endometriosis, together with selected associations involving TSH and hypo- or hyperthyroid phenotypes; reverse MR was null (21).

Overall, current epidemiological evidence supports a potential association between endometriosis and later thyroid-related morbidity (10–12). Within this literature, the most biologically plausible and recurrent signal involves thyroid autoimmunity rather than isolated biochemical thyroid dysfunction (10, 13, 14). Evidence is weaker for isolated biochemical thyroid dysfunction and remains conflicting for TRAb as an endometriosis-related biomarker (10, 15, 16, 19). The literature therefore supports further phenotype-specific investigation, but it does not establish a consistent causal relationship between thyroid-related phenotypes and endometriosis or endometriosis-related reproductive outcomes (10, 19). A structured summary and qualitative appraisal of the main evidence domains is provided in Table 1.

**Table 1:** Summary and appraisal of evidence linking thyroid-related phenotypes with endometriosis and reproductive outcomes.

Evidence domain	Thyroid-related phenotype(s)	Endometriosis or reproductive context	Main evidence	Interpretation for this review	Key limitation/certainty level
Retrospective and cross-sectional studies	TPOAb, TgAb; autoimmune thyroid disease	Endometriosis, especially ovarian endometriosis in selected clinical cohorts	Several studies reported higher thyroid autoantibody positivity in women with endometriosis or ovarian endometriosis. Some data suggested that combining thyroid autoantibodies with CA125 may improve discrimination in selected populations (13, 14).	This supports an autoimmune overlap signal, but it should not be interpreted as proof that thyroid autoantibodies are established diagnostic markers for endometriosis.	Low to moderate certainty. Findings are vulnerable to selection bias, retrospective exposure assessment, phenotype misclassification, population specificity, and incomplete control for infertility, autoimmune comorbidity, and healthcare utilization.
TRAb-focused studies	TRAb	Endometriosis biomarker research	Early work suggested elevated TRAb-related autoantibodies in women with endometriosis, whereas a later study using current clinical methods did not confirm elevated IgG TRAb (15, 16).	TRAb remains a research signal rather than a clinically actionable endometriosis biomarker.	Low certainty. Evidence is assay-dependent, limited by small or selected study populations, and inconsistent across testing methods. Independent validation is required before clinical interpretation.
Cohort and register-based studies	Functional thyroid disorders, thyroiditis, and other thyroid diagnoses	Long-term thyroid morbidity after endometriosis diagnosis	Long-term observational and register-based studies reported increased subsequent thyroid-related morbidity after endometriosis diagnosis (11, 12).	These studies provide temporal evidence, but residual confounding, surveillance bias, and healthcare-contact bias remain important limitations.	Moderate temporal value but not causal certainty. These studies improve chronological inference, but they remain vulnerable to residual confounding, differential thyroid testing, diagnostic ascertainment, and incomplete information on baseline thyroid status.
Infertility and assisted reproduction studies	TSH, thyroid autoimmunity, TPOAb, TgAb	Infertility, IVF/ICSI, and endometriosis-related reproductive outcomes	Thyroid autoimmunity is more common in infertile women overall. Studies of women undergoing assisted reproduction have examined coexisting endometriosis and thyroid autoimmunity, but independent effects on reproductive outcomes remain uncertain (17, 18, 29).	Reproductive relevance is plausible but likely subgroup-specific. Broad conclusions across all infertile or endometriosis populations should be avoided.	Context-specific certainty. Findings are limited by heterogeneous infertility causes, ovarian reserve, ART protocols, thyroid treatment status, outcome definitions, and confounding by indication.
Genetic overlap evidence	Thyroid signaling pathways and immune-related susceptibility	Shared biological susceptibility with endometriosis	Genetic overlap analyses have identified shared loci between endometriosis and immune-related conditions and have implicated pathways related to sex hormones and thyroid signaling (20).	These findings support shared susceptibility, but they do not prove that thyroid dysfunction causes endometriosis.	Moderate value for biological plausibility but low direct causal certainty. Genetic overlap may reflect pleiotropy, shared immune-endocrine architecture, or pathway-level convergence rather than direct thyroid-to-endometriosis causality.
Causal inference evidence	Genetically predicted TSH, FT4, thyroid dysfunction, and TPOAb	Overall and phenotype-specific endometriosis; endometriosis with infertility	An infertility-focused MR study did not support a simple causal effect of conventional thyroid function traits on overall infertility (19). A 2026 bidirectional MR study reported an inverse association of genetically predicted FT4 with overall endometriosis, selected subtype associations for TSH and hypo- or hyperthyroid phenotypes, and no reverse effect (21).	The evidence does not support a uniform thyroid hormone-driven causal model. The recent MR findings raise phenotype-specific hypotheses but are not sufficient for clinical inference.	Low-to-moderate causal-inference certainty. The new estimates require independent replication and remain limited by European ancestry, diagnosis-based subtypes, multiple comparisons, imprecision, and possible pleiotropy.

This table summarizes and appraises the main evidence domains discussed in the review. The certainty levels are qualitative judgments intended to reflect study design, phenotype specificity, consistency, and susceptibility to bias rather than formal GRADE ratings. The evidence should be interpreted as supporting association, biological plausibility, temporal co-occurrence, shared susceptibility, or causal uncertainty rather than establishing a simple thyroid hormone-driven causal pathway. Thyroid function traits and thyroid autoimmunity are presented as related but distinct constructs. ART, assisted reproductive technology; CA125, cancer antigen 125; FT4, free thyroxine; GRADE, Grading of Recommendations Assessment, Development and Evaluation; ICSI, intracytoplasmic sperm injection; IgG, immunoglobulin G; IVF, *in vitro* fertilization; MR, Mendelian randomization; TgAb, thyroglobulin antibodies; TPOAb, thyroid peroxidase antibodies; TRAb, TSH receptor antibodies; TSH, thyroid-stimulating hormone.

## Mechanistic basis and molecular signaling: established, suspected, and absent links

The mechanistic evidence should be interpreted according to the strength and location of biological support. At the first level, established endometriosis mechanisms provide the background against which any thyroid hypothesis must be evaluated. These include estrogen-dependent proliferation and progesterone resistance (3, 4). They also include impaired immune clearance, cytokine signaling, and complement activation (26–28), together with oxidative stress and metabolic remodeling (30–33). These mechanisms explain lesion survival and progression, but they do not establish thyroid involvement.

At the second level, thyroid-related reproductive mechanisms provide biologically plausible links. The hypothalamic-pituitary-thyroid axis interacts with the hypothalamic-pituitary-ovarian axis through changes in gonadotropin and prolactin secretion, ovarian steroidogenesis, sex-steroid availability, and endometrial receptivity (34, 35). Thyroid hormone signaling may also act locally through thyroid hormone receptors, TSH receptor signaling, deiodinase-mediated hormone activation or inactivation, and thyroid hormone transport. Experimental evidence suggests altered thyroid hormone handling in endometriotic cells, increased TSH receptor expression in ectopic endometrium, and proliferative or oxidative responses to thyroid-related stimulation (36). These findings provide a more direct mechanistic signal than epidemiological association alone. However, they remain preliminary and require independent replication across lesion phenotypes, menstrual-cycle phases, treatment states, and thyroid phenotypes.

The strongest direct lesion-level evidence comes from one translational study of human eutopic and ectopic endometrium, primary epithelial and stromal cells, and mouse models. Ectopic lesions showed higher thyroid-stimulating hormone receptor (TSHR) and thyroid hormone receptor alpha 2 (THRA2) expression. Iodothyronine deiodinase 2 (DIO2) was markedly reduced in eutopic and ectopic tissue, and monocarboxylate transporter 8 (MCT8; encoded by solute carrier family 16 member 2 [SLC16A2]) expression was lower in eutopic than ectopic or control tissue. These findings suggest reduced local T3 generation and altered thyroid hormone transport. TSH increased proliferation in control, eutopic, and ectopic cells. It also increased reactive oxygen species (ROS) in epithelial cells from all groups and in ectopic stromal cells. T4 increased proliferation and ROS in ectopic epithelial and stromal cells. T3 increased proliferation only in ectopic epithelial cells, but increased ROS in epithelial cells from all groups and in ectopic stromal cells. In mice, lesions were smaller during chemically induced hypothyroidism and larger in an autoimmune thyroiditis model with transient T3 and T4 elevations. However, a contribution from thyroid autoimmunity could not be excluded (36).

These experiments support a tissue-level thyroid-response phenotype, but they do not identify a complete signaling cascade. Based on receptor biology established outside endometriosis, candidate TSHR routes include stimulatory G protein alpha subunit (Gαs)–adenylyl cyclase–cyclic adenosine monophosphate and G protein alpha q/11 (Gαq/11)–phospholipase C signaling (37). Thyroid hormone action may also involve nuclear thyroid hormone receptor (TR)-mediated transcription. In non-endometriosis models, rapid signaling through integrin αvβ3 activates mitogen-activated protein kinase/extracellular signal-regulated kinase (MAPK/ERK), while T3 can activate a parallel phosphoinositide 3-kinase (PI3K) pathway (34, 35, 38). None of these downstream routes has been directly mapped, blocked, or rescued in human endometriotic cells. It is also unknown whether altered DIO2, THRA2, or MCT8 expression is a driver, a compensatory response, or a consequence of the lesion microenvironment. No lesion-specific study identified in this review has shown that correcting thyroid dysfunction, antagonizing TSHR, or restoring DIO2/TR signaling reduces human lesion growth. The direct mechanism therefore remains preclinical and incompletely specified rather than clinically established.

At the third level, the reviewed evidence is consistent with shared upstream susceptibility rather than a necessary thyroid-to-lesion pathway. The relevant evidence involves systemic immune dysregulation, autoimmune predisposition, endocrine-disrupting chemical exposure, genetic overlap, and epigenetic vulnerability, as summarized in Table 2. In this framework, thyroid autoantibodies may indicate thyroid-directed autoimmunity or broader immune susceptibility rather than mediate ectopic lesion implantation or survival. Direct antigenic cross-reactivity between thyroid antigens and endometriotic lesions was not demonstrated in the studies identified in this review. TRAb findings are also assay-dependent. An earlier study reported modest elevations, whereas a later study using current clinical assays did not confirm higher TRAb IgG levels (15, 16). TRAb should therefore not be interpreted as a validated diagnostic biomarker. The most defensible conclusion is not that thyroid dysfunction directly causes endometriosis. Instead, selected thyroid-related phenotypes may intersect with endometriosis through immune-inflammatory, endocrine, metabolic, environmental, and genetic pathways with different levels of evidential support. A pathway-specific evidence map is provided in Table 2.

**Table 2:** Mechanistic and molecular signaling pathways at the thyroid–endometriosis interface.

Mechanistic axis	Specific molecular sequence or components	Evidence status	Interpretation and principal missing evidence
Local thyroid hormone handling	TSHR↑ and THRA2↑; DIO2↓; altered MCT8/SLC16A2; reduced T4-to-T3 activation with relative local T4 accumulation (36)	Direct tissue-level, *in vitro*, and animal evidence from one translational study	The best direct mechanistic signal. Independent replication, lesion-subtype analysis, and causal perturbation of TSHR, DIO2, THRA2, or MCT8 are absent.
TSH/thyroid hormone signaling	TSH → proliferation and ROS in control, eutopic, and ectopic endometrial cells; T4 → proliferation and ROS in ectopic cells; T3 → ectopic epithelial-cell proliferation and broader epithelial ROS; candidate TSHR–adenylyl cyclase–cyclic adenosine monophosphate and phospholipase C routes; nuclear TR and integrin αvβ3-linked MAPK/ERK or PI3K routes (34–38)	Functional response demonstrated; downstream cascade not mapped	No receptor blockade, knockdown, rescue, phosphoproteomic mapping, or proof that these pathways mediate human lesion growth.
Immune tolerance and cellular clearance	Endometriosis-related immune abnormalities, including reduced NK-cell chemotaxis, cytokine dysregulation, and complement activation; thyroid autoantibodies as putative immune markers; thyroid-specific effects on NK-cell cytotoxicity, macrophage behavior, and immune-checkpoint signaling remain unproven (15, 16, 26–28)	Endometriosis immune biology established; thyroid-specific bridge suspected	No shared lesion antigen, antibody deposition, antigen-specific cross-reactivity, or proof that thyroid autoantibodies impair ectopic-cell clearance.
Cytokine and complement signaling	Peritoneal cytokine profiles and ERK/AKT/MAPK/STAT-family associations; IL-6-associated STAT3 activation; TGF-β-SMAD2/3 signaling (27, 39, 40); C3-C3a-C3aR mast-cell feed-forward loop (28)	Cytokine dysregulation and the C3 loop are supported; intracellular-route evidence varies; cross-condition role unproven	Direct evidence is strongest for STAT3, SMAD2/3, and the C3 loop; no thyroid-initiated lesion cascade has been demonstrated
HPT–HPO and sex-steroid crosstalk	Prolactin and gonadotropin changes; ovarian steroidogenesis; sex hormone-binding globulin; possible thyroid hormone-estrogen convergence outside endometriosis (34, 35, 43)	Physiologically plausible but indirect	No direct evidence that thyroid dysfunction causes ERβ dominance, PR-B loss, aromatase induction, or progesterone resistance in lesions.
Oxidative and metabolic remodeling	ROS; mitochondrial function; oxidative phosphorylation; PGK1-DDIT4, GLI1, and PRDX4 programs (30–33, 36)	ROS is a direct thyroid-related readout; other pathways are established only in endometriosis	The source and mediating role of ROS are unresolved. Thyroid perturbation has not been linked to the specified metabolic pathways.
Environmental and epigenetic convergence	BPA-WDR5/TET2-ERβ; broad PFAS/phthalate effects on endocrine signaling and oxidative or inflammatory processes (46, 48–50)	Shared-upstream susceptibility model	No evidence for a sequential exposure-to-thyroid-to-endometriosis mechanism; mixture effects, confounding, and reverse causation remain substantial.

This table separates molecular observations made directly in endometriotic tissue or models from pathways inferred from established endometriosis biology, reproductive thyroid physiology, or shared environmental susceptibility. Candidate downstream cascades are shown to define testable mechanisms; their inclusion does not imply that they have been demonstrated as thyroid-to-endometriosis mediators. AKT, protein kinase B; BPA, bisphenol A; C3, complement component 3; C3a, complement component 3a; C3aR, C3a receptor; DDIT4, DNA damage inducible transcript 4; DIO2, iodothyronine deiodinase 2; ERβ, estrogen receptor beta; ERK, extracellular signal-regulated kinase; GLI1, GLI family zinc finger 1; HPO, hypothalamic-pituitary-ovarian; HPT, hypothalamic-pituitary-thyroid; IL-6, interleukin-6; MAPK, mitogen-activated protein kinase; MCT8, monocarboxylate transporter 8; NK, natural killer; PFAS, per- and polyfluoroalkyl substances; PGK1, phosphoglycerate kinase 1; PI3K, phosphoinositide 3-kinase; PR-B, progesterone receptor B; PRDX4, peroxiredoxin 4; ROS, reactive oxygen species; SLC16A2, solute carrier family 16 member 2; SMAD2/3, SMAD family members 2 and 3; STAT, signal transducer and activator of transcription; STAT3, signal transducer and activator of transcription 3; T3, triiodothyronine; T4, thyroxine; TET2, tet methylcytosine dioxygenase 2; TGF-β, transforming growth factor beta; THRA2, thyroid hormone receptor alpha 2; TR, thyroid hormone receptor; TSH, thyroid-stimulating hormone; TSHR, thyroid-stimulating hormone receptor; WDR5, WD repeat domain 5.

## Immune dysregulation and autoimmune cross-talk

Immune dysregulation is one of the most plausible conceptual bridges between thyroid autoimmunity and endometriosis (7, 10). Autoimmune thyroid disease is characterized by thyroid-directed autoantibody production and immune-mediated thyroid dysfunction, particularly in Hashimoto's thyroiditis and Graves' disease (7, 8). Endometriosis is also shaped by immune dysfunction, including impaired clearance of ectopic endometrial cells, altered systemic and uterine immune populations, inflammatory immune-cell activation, and chronic inflammatory signaling (1, 5). Reduced peritoneal NK cell chemotaxis and immune checkpoint involvement have also been reported in endometriosis (6, 26). The coexistence of thyroid autoimmunity and endometriosis may therefore reflect shared immune susceptibility rather than a direct causal relationship (10, 13). To date, direct antigenic cross-reactivity between thyroid antigens and endometriotic lesions has not been established. Current evidence supports immune overlap rather than definitive autoimmune causality (10). At present, TPOAb and TgAb should be interpreted as markers of thyroid-directed autoimmunity or broader immune susceptibility rather than proven mediators of ectopic lesion survival (7, 10, 13). TRAb should be interpreted even more cautiously, because TRAb-focused studies remain assay-dependent and inconsistent (15, 16).

At the pathway level, peritoneal cytokine profiles and pathway analyses implicate ERK, protein kinase B (AKT), MAPK, and signal transducer and activator of transcription (STAT) family signaling. More direct endometriosis evidence supports interleukin-6 (IL-6)-associated signal transducer and activator of transcription 3 (STAT3) activation and transforming growth factor beta (TGF-β)-dependent SMAD family members 2 and 3 (SMAD2/3) signaling (27, 39, 40). Complement provides a separately studied amplification circuit. Locally produced complement component 3 (C3) is cleaved to the complement fragment C3a, which activates C3a receptor (C3aR)-positive mast cells and reinforces inflammatory-cell recruitment and lesion establishment (28). Reduced peritoneal NK-cell chemotaxis, cytokine dysregulation, and complement activation may each impair ectopic-cell clearance or sustain lesion-associated inflammation (26–28). However, the studies identified in this review do not show that thyroid autoantibodies initiate these routes, that they are selectively amplified in women with both conditions, or that pathway blockade interrupts a thyroid-endometriosis causal chain. Thyroid-related effects on NK-cell cytotoxicity, macrophage behavior, and immune-checkpoint signaling in lesions have also not been demonstrated. These pathways should therefore be described as candidate shared inflammatory routes rather than established cross-organ mechanisms.

Endometriosis is further characterized by phenotype-specific local and systemic inflammatory changes (1, 5). Peritoneal cytokine profiles may differ across endometriosis subphenotypes, suggesting that ovarian endometrioma and deep infiltrating disease are not immunologically identical (27). Systemic immune and tumor marker patterns may also be associated with disease severity and symptom burden (41). These inflammatory networks may contribute to lesion survival, angiogenesis, pain sensitization, and reproductive dysfunction (1, 3, 28). Because thyroid autoimmunity and endometriosis both involve immune-inflammatory contexts, chronic inflammation may provide a shared biological background linking endometriosis and thyroid abnormalities (7, 10). In this framework, thyroid autoimmunity is best interpreted cautiously as a potential marker of immune susceptibility unless direct tissue-level or antigen-specific evidence becomes available (7, 10, 13).

## Interaction between the HPT axis and the hypothalamic-pituitary-ovarian axis in uterine endocrine biology

The HPT axis and the hypothalamic-pituitary-ovarian (HPO) axis are closely connected (34). Thyroid dysfunction may affect gonadotropin secretion, prolactin release, ovarian steroidogenesis, sex-steroid availability, and reproductive endocrine balance (8, 34). TSH and thyroid hormone signaling may also participate in ovarian, endometrial, and luteal physiology, suggesting that thyroid-related signals can have reproductive effects beyond the thyroid gland (34, 35). Because endometriosis is estrogen-dependent, thyroid-related changes in reproductive endocrine balance could theoretically modify disease activity (1, 4, 34). However, this mechanism remains indirect and should not be interpreted as evidence that thyroid dysfunction initiates endometriosis (10, 19).

Thyroid hormones regulate metabolism, mitochondrial function, differentiation, and cell-cycle-related pathways in multiple tissues (34, 42). It is therefore biologically plausible that thyroid hormone signaling could influence reproductive tissues, including the endometrium, although direct evidence in endometriotic lesions remains limited (34, 35). Existing mechanistic studies in endometriosis have identified pathways involved in proliferation, migration, invasion, glycolysis, oxidative phosphorylation, and oxidative stress-related cellular behavior (30–33). These pathways have not yet been clearly linked to thyroid hormone signaling in endometriotic lesions. Future research should test whether thyroid hormone receptors, deiodinases, or thyroid hormone transporters are altered in eutopic endometrium or ectopic lesions. Experimental studies should also determine whether thyroid hormone exposure changes endometriotic stromal cell behavior.

Endometriosis is driven by estrogen-dependent proliferation and progesterone resistance (3, 4). Thyroid hormones may influence reproductive endocrine balance through effects on sex-steroid availability, ovarian function, and interactions with reproductive endocrine axes (8, 34). Experimental evidence from other estrogen-responsive tissues suggests that thyroid hormone signaling can interact with estrogen-mediated proliferation (43). However, extrapolation to endometriosis requires caution. Whether thyroid dysfunction contributes to progesterone resistance in endometriosis remains speculative and should be evaluated directly in eutopic endometrial and lesion tissues (3, 24, 25). Therefore, HPT-HPO axis interaction and sex-steroid availability should be considered plausible but indirect thyroid-related links, whereas thyroid receptor-, deiodinase-, or transporter-driven lesion biology remains unestablished.

Potential sex-steroid convergence is also unproven at the lesion level. Systemic thyroid dysfunction can alter prolactin, gonadotropin secretion, sex hormone-binding globulin, ovarian steroidogenesis, and sex-steroid availability, which could indirectly modify estrogen-dependent growth (34, 35). In a steroid receptor-positive breast cancer model, thyroid hormone enhanced estrogen-mediated proliferation (43). This finding supports possible endocrine convergence outside endometriosis, but it cannot establish a lesion-specific interaction between TR and the estrogen receptor (ER). Estrogen receptor beta (ERβ) dominance, progesterone receptor B (PR-B) loss, aromatase expression, and progesterone resistance are established lesion features (3, 4). The studies identified in this review do not show that thyroid dysfunction causes these changes. Receptor-occupancy, phosphoproteomic, and perturbation data are also lacking, so a specific TR–ER or TR–progesterone receptor pathway cannot be assigned.

## Shared metabolic and environmental contexts

Oxidative stress is a prominent feature of endometriosis and may contribute to inflammation, pain, and lesion persistence (1, 44). Endometriotic cells also show altered energy metabolism, including mitochondrial pathway involvement and increased glycolysis (30, 31). Because thyroid hormones regulate mitochondrial function and systemic energy metabolism, metabolic cross-talk between thyroid biology and endometriosis-related cellular metabolism is biologically plausible (42). However, a direct thyroid-driven metabolic mechanism in endometriotic lesions has not been demonstrated. This distinction is important because the current evidence supports biological plausibility rather than a confirmed thyroid-to-lesion metabolic pathway (10).

Among metabolic endpoints, ROS is currently the clearest direct readout of thyroid-related stimulation in endometriotic cells (36). However, its source, whether mitochondrial electron transport, nicotinamide adenine dinucleotide phosphate (NADPH) oxidases, or another pathway, was not examined in that study. Antioxidant rescue was also not performed, so ROS was not established as the mediator of proliferation. Connections to oxidative phosphorylation or programs involving phosphoglycerate kinase 1 (PGK1) and DNA damage inducible transcript 4 (DDIT4) described in endometriosis have not been tested under thyroid perturbation (30, 31). The same applies to programs involving GLI family zinc finger 1 (GLI1) and peroxiredoxin 4 (PRDX4) (32, 33). These networks should not be presented as thyroid-linked signaling pathways until direct perturbation studies are available.

Environmental exposures are discussed here not as independent causes of endometriosis, but as shared upstream factors that may simultaneously affect thyroid signaling, sex-steroid biology, immune-inflammatory tone, and uterine tissue regulation. Endocrine-disrupting chemicals may therefore provide a plausible exposure-based framework for linking thyroid-related endocrine disruption, immune-inflammatory pathways, and endometriosis. Bisphenol A (BPA) has been associated with enhanced endometrial stromal cell invasion and peritoneal endometriosis (45). Mechanistic studies also suggest that bisphenol A may affect estrogen receptor beta-related pathways in eutopic endometrium (46). Mixtures of non-persistent endocrine-disrupting chemicals have also been investigated in relation to endometriosis (47). In parallel, per- and polyfluoroalkyl substances (PFAS), phthalates, and other endocrine disruptors may affect thyroid-related endocrine signaling, hormone transport or metabolism, and immune-inflammatory responses (48–50). Endocrine-disrupting chemicals may also affect epigenetic regulation in uterine tissues, providing a possible link between environmental exposure, endocrine disruption, and reproductive tissue biology (51). These findings support a shared exposure framework rather than a direct thyroid-to-endometriosis pathway. Human evidence remains largely observational and is vulnerable to exposure measurement error, mixture complexity, residual confounding, and reverse causation (47–49). Overall, metabolic remodeling and oxidative stress are well-supported features of endometriosis, whereas thyroid-related metabolic effects and endocrine-disrupting chemicals are best interpreted as indirect or shared-upstream frameworks rather than confirmed thyroid-to-lesion mechanisms.

At the molecular level, BPA-associated endometrial evidence implicates WD repeat domain 5 (WDR5) and tet methylcytosine dioxygenase 2 (TET2) in ERβ regulation (46). Reviews of PFAS and phthalate exposure describe broad effects on endocrine signaling, hormone synthesis, transport or metabolism, and oxidative or inflammatory processes (48–50). However, these sources do not establish a lesion-specific molecular cascade or a human exposure-to-thyroid-to-endometriosis sequence. Endocrine-disrupting chemicals therefore remain a shared upstream model rather than a confirmed thyroid-mediated pathway.

## Genetic evidence and causal inference

Genetic susceptibility may contribute to endometriosis and may also implicate thyroid-related biological pathways (10, 20). Genetic overlap analyses have identified shared loci between endometriosis and immune-related conditions and have implicated pathways related to sex hormones and thyroid signaling (20). These findings support the possibility of shared biological susceptibility, but they do not prove that thyroid dysfunction causes endometriosis or that endometriosis causes thyroid disease (10, 20). Epigenetic mechanisms may also link environmental exposures with uterine endocrine regulation, inflammatory signaling, and endometrial disease biology (51). Genetic and epigenetic findings therefore support shared susceptibility and pathway convergence, but they do not establish that thyroid dysfunction directly initiates or maintains endometriotic lesions. A recent bidirectional MR analysis adds direct but preliminary genetic evidence: several associations were phenotype-specific, and the authors called for independent replication (21).

The thyroid-endometriosis relationship is difficult to interpret because both conditions are common, multifactorial, and influenced by reproductive, immune, genetic, environmental, and healthcare-related factors (10, 11). Potential confounders may include age, BMI, parity, infertility, autoimmune comorbidity, hormonal treatment, thyroid medication, iodine status, environmental exposures, socioeconomic factors, and healthcare utilization (10, 11). Cross-sectional studies can identify co-occurrence but cannot establish temporal sequence. Case-control studies may generate hypotheses but are vulnerable to selection bias, recall bias, and retrospective exposure assessment. Cohort studies improve temporal inference but remain vulnerable to residual confounding and surveillance bias (11, 12).

MR can reduce confounding and reverse causation by using genetic variants as instrumental variables, but its validity depends on the relevance, independence, and exclusion-restriction assumptions (52). It also requires accurate phenotype definitions, sufficient instrument strength, adequate statistical power, and careful assessment of horizontal pleiotropy (52, 53). An infertility-focused MR study did not support a simple effect of conventional thyroid function traits on overall infertility (19). A 2026 bidirectional two-sample MR study reported an inverse association between genetically predicted FT4 and overall endometriosis, together with selected associations for TSH and hypo- or hyperthyroid phenotypes; reverse MR was null. Interpretation is limited by European ancestry, diagnosis-based subtypes, multiple comparisons, imprecision, and residual pleiotropy (21). Together, these findings do not establish a uniform thyroid-to-endometriosis causal pathway, but they leave open smaller or phenotype-specific effects involving thyroid autoimmunity, ovarian endometrioma, deep infiltrating disease, endometriosis-associated infertility, or pain-dominant phenotypes (10, 17, 19). Current evidence is therefore best interpreted through four parallel, non-mutually exclusive explanatory models. First, thyroid-related phenotypes may contribute to selected endometriosis or infertility phenotypes (10, 17, 19). Second, endometriosis-related inflammation, treatment, or healthcare contact may increase the likelihood of later thyroid diagnosis (10–12). Third, both conditions may arise from shared immune, endocrine, genetic, environmental, or epigenetic susceptibility (20, 47, 51). Fourth, part of the observed association may reflect differential thyroid testing, diagnostic ascertainment, or surveillance bias (10, 11). Available evidence is insufficient to select one model as dominant across all populations. Future studies should distinguish thyroid hormone traits from autoimmune thyroid traits and should avoid treating endometriosis as a single homogeneous outcome (10, 19, 20).

## Clinical and translational implications

**Figure 2:**
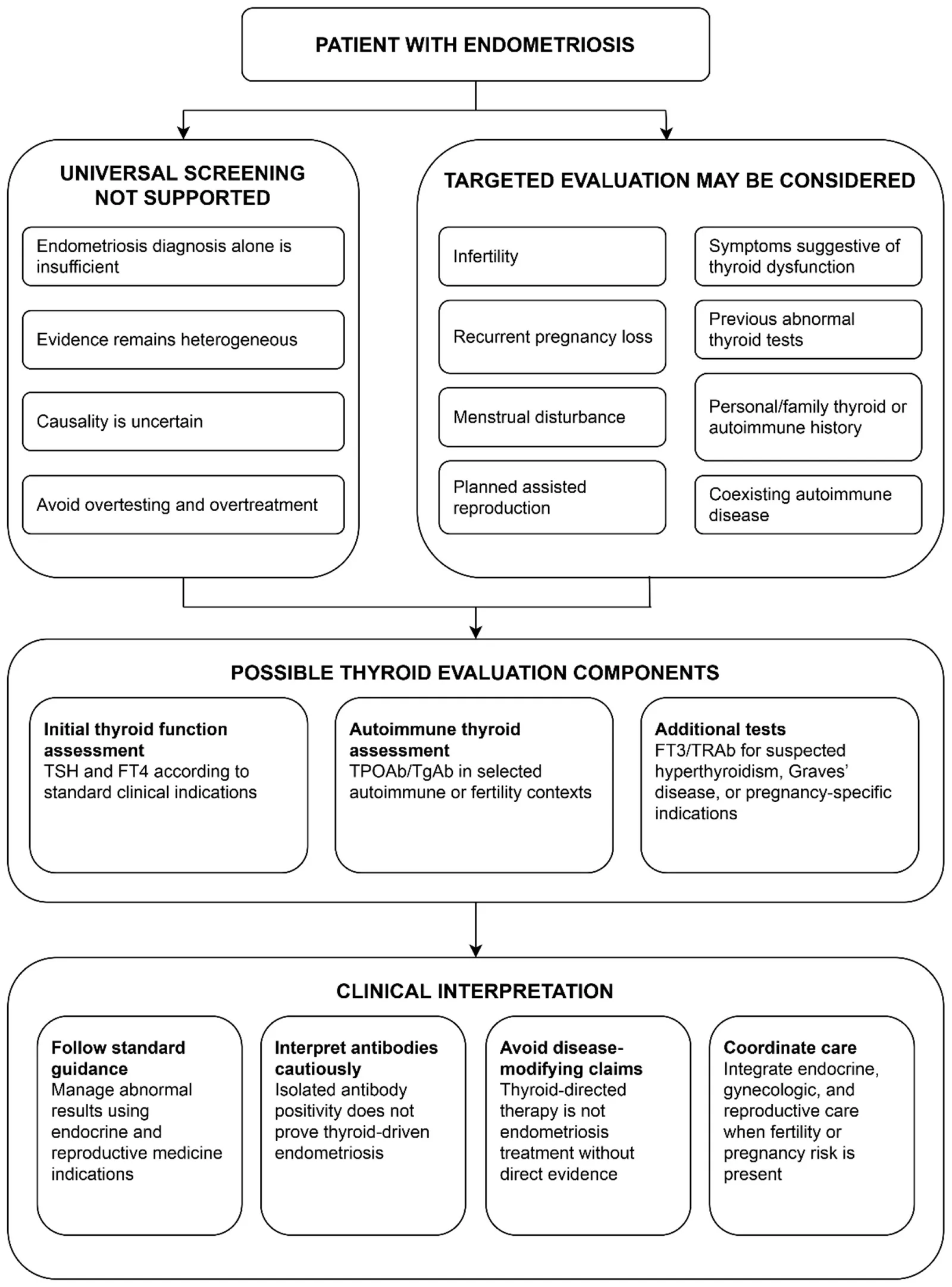
Proposed interpretive framework for targeted thyroid evaluation in selected patients with endometriosis. This figure outlines a phenotype-aware framework for targeted thyroid evaluation in selected patients with endometriosis. Current evidence does not support universal thyroid screening solely because endometriosis is present. Thyroid testing should instead be considered when established endocrine, reproductive, pregnancy-related, or autoimmune indications are present, such as infertility, recurrent pregnancy loss, menstrual disturbance, planned assisted reproductive treatment, symptoms suggestive of thyroid dysfunction, previous abnormal thyroid tests, or personal or family history of thyroid or autoimmune disease. TSH and FT4 remain first-line tests for thyroid function assessment. TPOAb and TgAb may be considered when autoimmune thyroid disease is suspected, whereas TRAb testing should generally be reserved for suspected Graves' disease or specific pregnancy-related indications. This figure is intended as an interpretive framework and does not replace existing endocrine, reproductive medicine, or pregnancy-specific clinical guidelines. ART, assisted reproductive technology; FT4, free thyroxine; TgAb, thyroglobulin antibodies; TPOAb, thyroid peroxidase antibodies; TRAb, TSH receptor antibodies; TSH, thyroid-stimulating hormone.

Current evidence does not justify universal thyroid screening solely on the basis of an endometriosis diagnosis (10, 19). Endometriosis guidelines focus on diagnosis, pain management, infertility care, and hormonal or surgical treatment, but they do not recommend thyroid testing as a routine endometriosis-specific intervention (2). Therefore, thyroid testing should not be framed as routine screening for endometriosis itself. A more cautious and evidence-aligned approach is to evaluate thyroid function and thyroid autoimmunity when standard endocrine, reproductive, pregnancy-related, or autoimmune indications are present, such as infertility, recurrent pregnancy loss, menstrual disturbance, planned assisted reproductive treatment, symptoms suggestive of thyroid dysfunction, previous abnormal thyroid results, or personal or family history of thyroid or autoimmune disease (7–9, 54, 55). In this framework, the purpose of thyroid evaluation is to identify clinically relevant thyroid disease in selected patients rather than to treat thyroid testing as an endometriosis-specific diagnostic or disease-modifying intervention. A proposed framework for targeted thyroid evaluation in selected patients with endometriosis is summarized in Figure 2.

TSH and FT4 remain appropriate first-line tests for thyroid function in reproductive and assisted reproduction settings (8, 9). TPOAb and TgAb may be considered when autoimmune thyroid disease is suspected or when fertility-related risk assessment is clinically relevant (7, 8). TRAb testing should generally be reserved for suspected Graves' disease or specific pregnancy-related indications (23, 54, 55). Its role as an endometriosis biomarker remains unvalidated because early TRAb signals have not been consistently confirmed by current clinical methods (15, 16). Thyroid dysfunction should be managed according to established endocrine and reproductive medicine principles (8, 9, 54, 55). At present, thyroid treatment should not be presented as a disease-modifying therapy for endometriosis unless standard thyroid-related indications are present (2, 3, 10).

## Future directions

Future studies should use standardized definitions of both endometriosis and thyroid disease (2, 10). Surgically confirmed endometriosis should be distinguished from self-reported, symptom-based, or diagnosis-code-based disease (2, 12). Lesion phenotypes should also be analyzed separately, because ovarian endometrioma, deep infiltrating endometriosis, and other phenotypes may differ in endocrine, inflammatory, and immune profiles (24, 25, 27). Thyroid phenotypes should distinguish overt hypothyroidism, subclinical hypothyroidism, and hyperthyroidism (8, 9, 22, 23). Autoimmune and structural thyroid phenotypes, including Hashimoto's thyroiditis, Graves' disease, thyroiditis, nodular disease, and isolated thyroid antibody positivity, should also be analyzed separately (7, 8, 10). Functional thyroid disorders should also be separated from thyroid autoimmunity, because thyroid hormone abnormalities and thyroid antibody positivity are related but non-identical constructs (7, 10).

Prospective cohorts are needed to clarify temporal relationships (11, 12). Repeated measurements of TSH, FT4, FT3, TPOAb, TgAb, and TRAb before and after endometriosis diagnosis would help determine whether thyroid abnormalities precede disease onset, emerge after diagnosis, or fluctuate with disease activity (8, 10). Future cohorts should ideally capture endometriosis phenotype, diagnostic method, pain and infertility status, hormonal treatment, thyroid medication, iodine-related information, BMI, smoking status, autoimmune comorbidity, environmental exposures, and healthcare utilization (10, 11, 47).

Mechanistic studies should examine thyroid hormone signaling and thyroid autoimmunity in eutopic endometrium, ectopic lesions, peritoneal fluid, immune cells, and animal models (5, 10, 34, 35). Relevant targets include thyroid hormone receptors, deiodinases, thyroid hormone transporters, estrogen signaling, and progesterone receptor pathways (3, 10, 35). Immune and inflammatory targets should include cytokine networks, NK cell function, complement activation, oxidative stress markers, and broader immune-cell activation (26–28, 44). These studies should test whether thyroid-related pathways are directly involved in endometriotic lesion biology or instead reflect systemic immune-endocrine dysregulation (10, 19).

Finally, causal inference should be expanded through bidirectional MR, multivariable MR, genetically informed cohort studies, and target trial emulation (52, 53, 56). These approaches should distinguish conventional thyroid function traits from autoimmune thyroid traits and should avoid treating endometriosis as a single homogeneous outcome (10, 19, 20). Integrating genetic, epigenetic, immune, endocrine, and environmental exposure data may help identify shared pathways linking thyroid dysfunction, thyroid autoimmunity, and endometriosis (20, 51). Key research priorities and methodological recommendations are summarized in Table 3.

**Table 3:** Future research priorities for the thyroid–endometriosis interface.

Research area	Current limitation	Recommended approach	Expected contribution
Endometriosis phenotyping	Self-reported, symptom-based, and diagnosis-code-based definitions may introduce misclassification. Lesion phenotypes are often pooled.	Separate surgically confirmed disease from self-reported or code-based disease. Analyze ovarian endometrioma, deep infiltrating endometriosis, superficial disease, pain-dominant phenotypes, and infertility-associated phenotypes separately (2, 12, 27).	Improve outcome specificity and reduce dilution from biologically heterogeneous endometriosis phenotypes.
Thyroid phenotyping	Thyroid hormone traits, thyroid dysfunction, thyroid autoimmunity, and diagnosis-based thyroid disease are often combined as a single exposure construct.	Distinguish TSH, FT4, FT3, TPOAb, TgAb, TRAb, overt hypothyroidism, subclinical hypothyroidism, hyperthyroidism, Hashimoto's thyroiditis, Graves' disease, thyroiditis, and isolated antibody positivity (7–10).	Reduce exposure misclassification and clarify whether the association is driven by thyroid autoimmunity, biochemical dysfunction, or diagnosis-based disease.
Prospective cohorts	Temporal sequence remains uncertain, and most studies cannot separate pre-existing thyroid abnormalities from thyroid changes after endometriosis diagnosis.	Use repeated measurements of TSH, FT4, FT3, TPOAb, TgAb, and TRAb before and after endometriosis diagnosis. Collect data on age, BMI, iodine-related information, smoking, hormonal treatment, thyroid medication, autoimmune comorbidity, environmental exposures, and healthcare utilization (8, 10, 11, 47).	Clarify whether thyroid abnormalities precede endometriosis, follow endometriosis, fluctuate with disease activity, or reflect shared upstream factors.
Mechanistic and translational studies	Direct tissue-level evidence linking thyroid pathways to endometriotic lesion biology remains limited.	Examine thyroid hormone receptors, deiodinases, thyroid hormone transporters, estrogen signaling, and progesterone receptor pathways in eutopic endometrium, ectopic lesions, and animal models. Also assess cytokine networks, NK-cell function, complement activation, oxidative stress, and broader immune-cell activation in peritoneal fluid and immune-cell compartments (26, 28, 34, 35).	Move from biological plausibility to testable mechanisms and identify whether thyroid-related pathways are lesion-level drivers or systemic markers.
Environmental and epigenetic studies	Human exposure evidence is limited by measurement error, mixture complexity, residual confounding, and reverse causation.	Use repeated exposure measurements, mixture modeling, epigenetic endpoints, and tissue-specific molecular analyses to evaluate BPA, PFAS, phthalates, and other endocrine-disrupting chemicals (45–51).	Clarify whether environmental exposures act as shared upstream factors affecting thyroid regulation, immune-inflammatory pathways, and endometriosis biology.
Causal inference and trial emulation	One recent bidirectional MR study reported phenotype-specific associations, but independent replication, colocalization, multivariable analyses, and instruments for autoimmune thyroid traits remain limited.	Use independent bidirectional and multivariable MR, colocalization, genetically informed cohort designs, and target trial emulation. Separate conventional thyroid function traits from autoimmune thyroid traits and avoid treating endometriosis as a homogeneous outcome (19–21, 52, 53, 56).	Test whether reported subtype associations replicate and separate causality from shared susceptibility, surveillance bias, confounding, and horizontal pleiotropy.

This table summarizes research priorities derived from the evidence gaps discussed in the review. The proposed approaches are intended to improve phenotype specificity, temporal inference, mechanistic validation, environmental exposure assessment, and causal interpretation. BMI, body mass index; BPA, bisphenol A; FT3, free triiodothyronine; FT4, free thyroxine; MR, Mendelian randomization; NK, natural killer; PFAS, per- and polyfluoroalkyl substances; TgAb, thyroglobulin antibodies; TPOAb, thyroid peroxidase antibodies; TRAb, TSH receptor antibodies; TSH, thyroid-stimulating hormone.

## Conclusions

Thyroid dysfunction, thyroid autoimmunity, and endometriosis are clinically relevant conditions in reproductive-aged women, and accumulating evidence suggests a potential but heterogeneous association among them (10, 11, 13). For reproductive biology and endocrinology, the strongest rationale lies in thyroid autoimmunity, immune dysregulation, chronic inflammation, uterine endocrine biology, and reproductive endocrine cross-talk rather than isolated thyroid hormone abnormalities alone (7, 10, 34).

Current evidence remains insufficient to establish a simple causal relationship between thyroid-related phenotypes and endometriosis or endometriosis-related reproductive outcomes (10, 19). A recent bidirectional MR analysis reported several phenotype-specific associations, but the estimates do not yet establish a reproducible or clinically actionable causal effect (21). The thyroid-endometriosis association may reflect shared immune-endocrine susceptibility, environmental exposures, genetic or epigenetic vulnerability, bidirectional biological interactions, differential thyroid testing and diagnostic ascertainment, or a combination of these factors (11, 20, 51). This uncertainty is clinically important. Thyroid autoimmunity may help identify selected reproductive-risk phenotypes, but it should not be treated as proof of thyroid-driven endometriosis (7, 10).

Universal thyroid screening for all women with endometriosis is not currently justified (2, 10). Thyroid evaluation should instead be targeted to established endocrine, reproductive, pregnancy-related, or autoimmune indications, such as infertility, recurrent pregnancy loss, menstrual disturbance, planned assisted reproduction, symptoms suggestive of thyroid dysfunction, previous abnormal thyroid tests, or autoimmune comorbidity (7–9, 55). Future research should move beyond broad associations toward phenotype-specific, longitudinal, mechanistically informed, and causally robust studies (10, 52, 53). Such work is needed to clarify whether thyroid-related pathways are clinically useful markers, modifiable contributors, or parallel manifestations of systemic reproductive immune-endocrine dysregulation (10, 19).
